# Urban Seismology: on the origin of earth vibrations within a city

**DOI:** 10.1038/s41598-017-15499-y

**Published:** 2017-11-10

**Authors:** Jordi Díaz, Mario Ruiz, Pilar S. Sánchez-Pastor, Paula Romero

**Affiliations:** Institute of Earth Sciences Jaume Almera. Consejo superior de Investigaciones Científicas (ICTJA-CSIC), Barcelona, Spain

## Abstract

Urban seismology has become an active research field in the recent years, both with seismological objectives, as obtaining better microzonation maps in highly populated areas, and with engineering objectives, as the monitoring of traffic or the surveying of historical buildings. We analyze here the seismic records obtained by a broad-band seismic station installed in the ICTJA-CSIC institute, located near the center of Barcelona city. Although this station was installed to introduce visitors to earth science during science fairs and other dissemination events, the analysis of the data has allowed to infer results of interest for the scientific community. The main results include the evidence that urban seismometers can be used as a easy-to-use, robust monitoring tool for road traffic and subway activity inside the city. Seismic signals generated by different cultural activities, including rock concerts, fireworks or football games, can be detected and discriminated from its seismic properties. Beside the interest to understand the propagation of seismic waves generated by those rather particular sources, those earth shaking records provide a powerful tool to gain visibility in the mass media and hence have the opportunity to present earth sciences to a wider audience.

## Introduction

The center of a large European city is clearly not the most suitable location for a seismic station, as the high vibration levels resulting from traffic, machinery, electric power lines and people moving around mask most of the signals generated by natural seismicity. Nevertheless, urban seismology has become an active research field in the last decades, mostly to characterize the subsurface structure and hence improve seismic microzonation and seismic risk management in populated areas (e.g.^1^ and references hereby). The interest on seismic signals of non-natural origin has boosted in the last decade following the increasing number of continuously recording digital broad-band seismic stations and the development of techniques to use ambient noise to retrieve tomographic images at different scales e.g. ref.^2^. Earth shaking from traffic and railways has been used to extract shear waves and construct stacked seismic images or to invert surface wave travel times^3–5^. Seismic sensors have been proposed as an efficient way to detect and characterize moving vehicles on roadways^6^. High density deployments involving several thousands of sensors have allowed not only to image seismic wave propagation through urban environments, but also monitor train and traffic circulation, aircraft landings and other man-made sources of shaking^7^. Most of those studies have been carried on using short period or accelerometric sensors, as they were focused in the analysis of high frequency signals. However, some studies based on seismic data recorded by broad-band seismometers deployed in urban environments have been published in the last years. Groos and Ritter^8^ analyzed data from a broad-band network installed at Bucharest to investigate the source of seismic signal across the different frequency bands. They conclude that human activity is the dominant source for frequencies below 0.1 Hz and above 1 Hz, although contribution from wind is observed in the 0.6–1.2 Hz band for velocities exceeding 3–4 m/s^9^. Boese *et al*.^10^ have investigated the seismic spectrum recorded by borehole seismometers installed at Auckland, New Zealand. The authors identified signals generated by traffic and train passages in the 1–35 Hz and 8–35 Hz bands, as well as elevated noise levels during the 2011 Rugby World Cup. Recently, Green *et al*.^11^ have analyzed a network of 5 broad-band seismometers operated in central London during about one month. Again, human generated signals dominate most of the seismic spectra, except for the microseismic secondary peak (0.16–0.5 Hz) and vibrations related to subway circulation are observed both at high and low frequencies.

**Figure 1:**
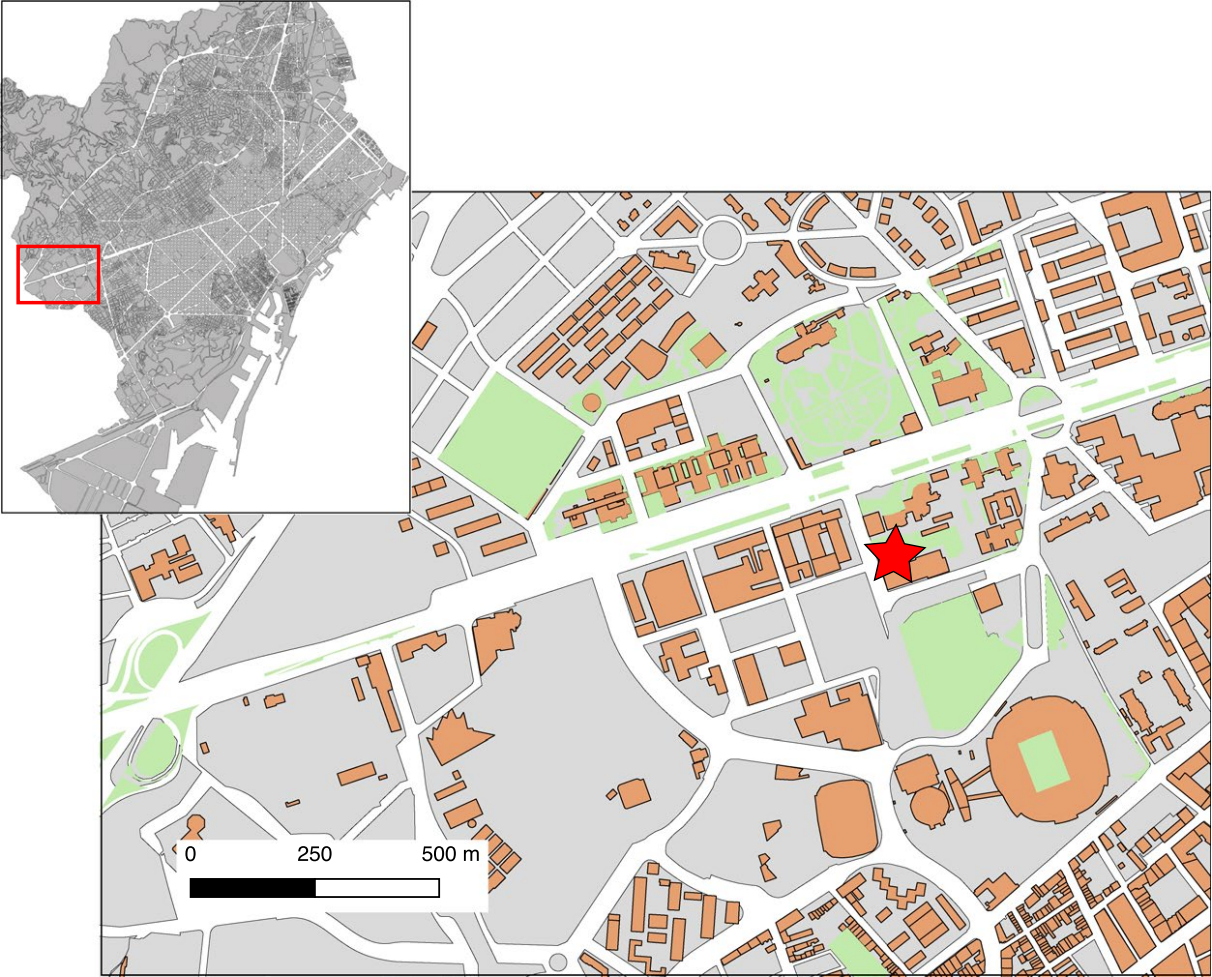
Detailed map of the ICTJA-CSIC site. Red star shows the building location. The onset shows the area (identified by a red box) in a general plan of Barcelona. This map has been produced using QGIS 2.18 (https://www.qgis.org/) and is based on the digital geographic information from *CartoCiudad* assigned by © *Instituto Geográfico Nacional* (http://www.cartociudad.es/visor/).

A secondary motivation to maintain a seismic station in an urban environment is science dissemination, as it can provide eye-catching visualization of distant earthquakes shaking the building, a fact usually surprising non-seismologists. The ICTJA-CSIC research institute, located in downtown Barcelona (Fig. 1), maintains since early 2011 a SEP educational horizontal seismometer installed near the building entrance, allowing to present to visitors the principles of operation of the basic instrument in Earth Sciences and to put on evidence the huge energy delivered by earthquakes, which often overprints the shaking related to traffic and human activities. Since early 2015, an additional seismic station equipped with a three component broad-band sensor, coded as ICTJA, has been installed at the basement of the building and connected to a Seiscomp server to offer real-time monitoring of the seismic activity through the institute webpage (http://www.ictja.csic.es). In this case, we use a Nanometrix Trillium Compact sensor, a broad-band seismometer with flat response extended till periods of 120 s. The sampling has been fixed to 250 Hz, to allow the detection of high frequency seismic signals. Although the data were not intended to be exploited scientifically, looking at them has revealed to be an interesting approach. In this contribution we document the origin of the features identified in the records, mostly related to man-made activities.

## An overview of the seismic spectra

**Figure 2:**
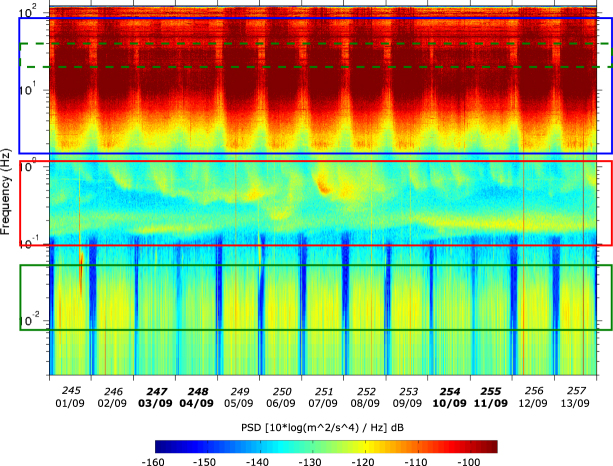
Spectrogram of the vertical seismic acceleration during the time interval 01/9/2016–13/9/2016. Red box shows the frequency range dominated by ocean activity, blue box shows the band dominated by traffic and green boxes show the low frequency (continuous line) and high frequency (dashed line) bands associated to subway activity. Week-ends are shown by bold dates. Color scale is expressed in dB referred to 1 (m^2^/s^4^)/Hz.

It has been widely documented that the seismic signal has different sources and generation mechanisms at different frequency bands^1,12^. One of the best tools to illustrate those differences are the spectrograms, a representation in which the seismic acceleration is decomposed to get the evolution of its power spectral density in function of time and frequency, expressed in dB and relative to a reference value of 1 (m^2^/s^4^)/Hz (Fig. 2). At high frequencies, the seismic signal is dominated by man-made activities, as denoted by the large daytime/nighttime variation, with large energy during working hours and much less during nighttime and week-ends. The 0.04–1 Hz band, known as the microseismic band, concentrates the largest seismic energy in quiet broad-band stations distributed worldwide and its origin is related to waves in the ocean that couples to elastic waves in the seabed and then propagates primarily as surface waves in the Earth^13,14^. The microseismic band can be recognized at ICTJA, although it is less prominent than for typical. The energy at frequencies below 0.05–0.1 Hz is dominated by day-night variations which are unrelated with natural source signals and will be discussed later. Within 0.1 and 0.25 Hz the time variations can be related to oceanic wave height changes in the Atlantic and Mediterranean basins. Above 0.25 Hz, the energy variations have a clearly different, more patched aspect, a feature probably related to local wind interacting with buildings and structures nearby the station^15^.

## Discussion on the origin of the observed seismic signals

### Traffic monitoring

**Figure 3:**
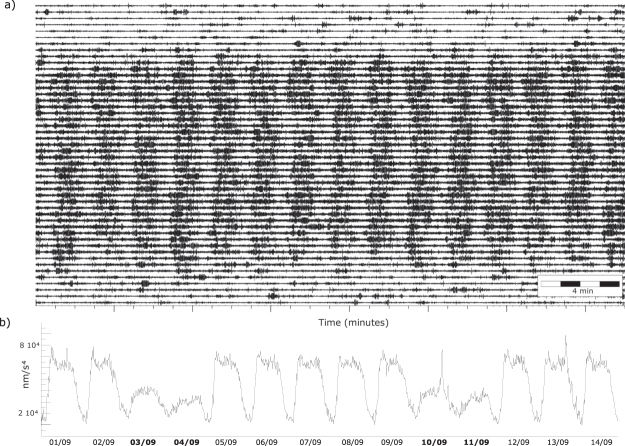
Seismic signal generated by road traffic. (**a**) Vertical component of the seismic acceleration band-pass filtered between 8 and 12 Hz. The image shows the complete record for day 09/07/2016, with each line displaying 30 minutes of data. The noise banding reflects the traffic along Av. Diagonal, regulated using a traffic light. (**b**) Envelope of the vertical component of acceleration, filtered between 8 and 12 Hz and decimated to 1 sample every 10 minutes. Night/day and working day/week-end variations are clearly evidenced. Note the rush hour in working day’s morning and the lower amplitude during Saturdays and Sundays (labeled in bold). Narrow peaks on days 10 and 13 are associated to traffic increase before and after FC Barcelona football games.

The spectrogram of the recorded signal shows a large energy variation between daytime and nighttime, as well as between working and holiday days, for frequencies above 1 Hz. This pattern can be identified for frequencies above 1, with maximum energy between 8 and 35 Hz and denotes the anthropogenic origin of the signal (Fig. 2). Exploring the signal in narrower bandwidths, a close inspection of the time series once band-pass filtered between 8 and 12 Hz shows that during daytimes, from around 06:30 to 22:30 local time, the signal is dominated by a regular pattern of low and high energy cycles about 2 minutes long (Fig. 3a). After discarding potential sources as engines located close to the recorder (air-conditioning units, fridges…), we related this signal pattern to the traffic activity in the nearby Av. Diagonal, one of the main traffic entrances to Barcelona city and located at about 150 m North of our laboratory (Fig. 1). This large avenue is crossed by a street deserving only local traffic and hence the traffic lights are unevenly regulated. We have verified that traffic along Av. Diagonal is open for 90 s, while vehicles using the transversal street have 30 s to cross, a sequence adjusting nicely the 2 minutes cycle observed in the band-pass filtered seismograms (Fig. 3a).

Figure 3b show the time evolution of the seismic noise during a two weeks period. During daytime, amplitudes at working days roughly doubles those observed during week-ends. Nighttime amplitude is about 4 times smaller than during daytime and Friday and Saturday nights show larger amplitudes than the rest of nights, reflecting higher nocturnal activity in the city during week-ends. Working days show a clear amplitude peak between 5:00 and 8:30 UTC (6:00–9:30 local time), related to traffic increase during rush hours. On contrary, an evening rush hour is not easy to identify. Sunday afternoons display a relative peak associated to cars returning to the city after week-end break. The narrow and energetic peaks observed the 10^th^ and 13^th^ September are related to traffic increases before and after two football games in the nearby FC Barcelona Stadium. Therefore, it is clearly documented that a seismometer in the city can be used to monitor traffic levels, even if located 150 m away from the monitored road.

### Vibrations induced by subway trains

Different studies have analyzed the seismic noise generated by railway traffic and proposed models to predict the induced vibrations^16–21^. Seismic sensors have also been used to monitor the vibrations generated by subway trains circulating close to historical buildings, as the Cologne Cathedral in Germany or the Bell Tower in Xi’an (China)^22,23^. Most of those studies use data recorded directly beside the railway and focus on the high frequency band of the seismic records. In our case, the high sensibility of the broad-band instrument allows to record individual passages of tube trains along a subway line running beneath Av. Diagonal, at a distance of about 150 m of the recording site.

**Figure 4:**
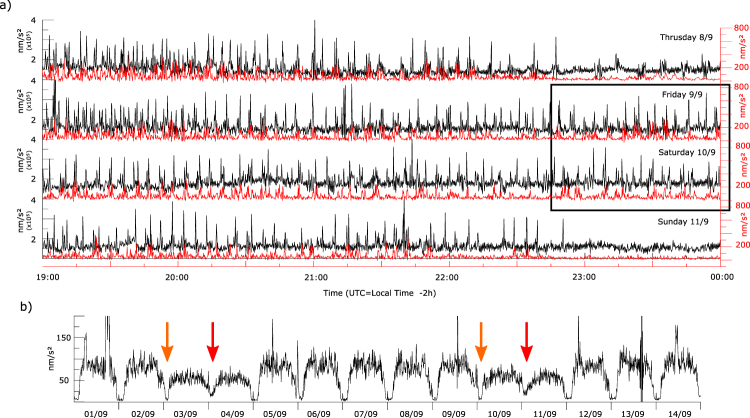
Seismic signal related to subway activity. (**a**) Envelope of the vertical acceleration for the interval 19:00–24:00 UTC (21:00–02:00 Local time) from Thursday to Sunday, filtered in the 20–40 Hz (black line) and the 0.008 and 0.05 Hz (red line) bands and decimated to 1 sample every 10 seconds. Late service during Friday and Saturday nights are identified by a box. (**b**) Envelope of acceleration filtered between 0.008 and 0.05 Hz and decimated to 1 sample every 10 minutes. Friday and Saturday nights are identified with orange and red arrows and clearly differ from the rest of the week. Large peak on day 01/09 correspond to a 7.0 earthquake with epicenter in New Zealand. Other peaks are related to local noise sources.

The metro system at Barcelona works from 05:00 to 00:00 (local time) Monday to Thursday, 05:00–02:00 on Friday and continuously from 05:00 Saturday until 00:00 on Sunday. This rather complex activity pattern allows an easy comparison with the signal amplitude variations in the seismic data. The train passage interval during peak hours is reported to be close to 200 s and increases during valley hours till 5–7 minutes. Temporal changes in the properties of the recorded seismic data consistent with the subway time activity cycles are identified in two frequency bands (Fig. 4a). At high frequencies, individual passages of subway trains are recognized in the 20–40 Hz band, coherently to observations described in different cities worldwide, as Cologne^22^, Long Beach^7^, Beijing^24^, London^11^ or Auckland^10^. Figure 4a shows a clear relationship between the observed peaks and train passages, with in the passage interval increasing progressively after 22:00 local time and during Saturdays and Sundays. As the trains circulate in two senses and we can not discriminate among them, it is difficult to fix the passage interval, but the data suggest that it is around 4 minutes during working hours and increase to around 8 minutes during night, consistently with the reported timetable.

The seismic signal related to subway trains can also be identified in the 0.008–0.05 Hz band (periods of 20–125 s), in the low frequency side of the microseismic peak. This signature can be observed in the temporal series of the filtered data (Fig. 4b) and in the spectrogram (Fig. 2), that mimics the subway time activity cycles, with shorter periods of low noise on Friday nights and no low noise intervals during Saturday nights. As in the case of high frequencies, the envelope of the vertical seismic acceleration allows to identify individual train passages (Fig. 4a). The peaks in this band have less amplitude and are somehow blurred, probably because the contribution of other sources is more relevant. Sheen *et al*.^25^ have reported a similar observation near subway lines at Seoul and some more sites sparse worldwide (Palisades, New York; Memphis, Tennessee; and Trieste, Italy) without providing a theoretical explanation for them. Green *et al*.^11^ reported a short duration, low amplitude displacement signature in a seismic station located almost directly above subway tunnels. This signature was interpreted as a quasi-static response to the train passage, following the modeling proposed by Yang *et al*.^26^. Under this hypothesis, the signal will correspond to the tunnel deformation generated by the weight of the train. However, it seems unlike that the observations in our station, located at about 150 m of the tunnels, could be related to those tiny tunnel deformations. Wielandt^27^ has reported that broad-band seismometers are sensitive to variations of magnetic field in the ground, in particular in urban environments, which may explain the seismic low frequency signals. DC electric railways (subway, tramway) produce magnetic fields both from the intended traction currents and from the stray currents leaking to the Earth, although the first ones are only relevant on or near the train^28^. Estimating the intensity of the magnetic field variations induced by railways is complex and depend on multiple parameters^29^. Hence, further developments will be needed to assess if magnetic field perturbations can explain our observations. Hereof, at this point we favor the hypothesis of magnetic field variations due to stray currents to explain the long period seismic signals related to subway activity detected at relatively far distance.

### Vibrations related to Rock concerts

One rather unusual signal observed in our seismic data corresponds to the earth vibrations recorded during rock concerts performed at the FC Barcelona stadium, located about 500 m southeastwards of the ICTJA building (Fig. 1). Some precedents do exist on this kind of seismic recordings; Erlingsson and Bodare^30^ studied the vibrations induced by rock concerts in Ullevi Stadium (Gothenburg, Sweden). Green and Bowers^31^ have investigated the seismic records generated by an electronic dance music festival in England, observing high amplitudes within the 2–3 Hz band that remain stable over periods of hours and were related to the vibration of the festival loudspeakers coupling into the ground at the festival site and then propagating as surface waves. The Institute of Geological and Nuclear Sciences of Australia (GEONET) reported the seismic recordings during a Foo Fighters concert in Auckland on December 2011 and during an AC/DC concert on December 2015 (http://geonet-shakennotstirred.blogspot.com.es/). Bertero *et al*.^32^ recorded using accelometric sensors the vibrations generated by rock concerts held at River Plate Stadium in Buenos Aires (Argentina) on neighborhood buildings and interpreted the signal as Rayleigh waves resulting from the coordinate jumping of spectators, with a first harmonic close to 2.1 Hz. Finally, Denton^33^ has analyzed seismic records during two of the songs of a Madness concert in the Reading Festival in 2011, reporting distinct at frequencies of 2.08 and 2.5 Hz, with harmonics at 4.16 and 5.0 Hz respectively.

**Figure 5:**
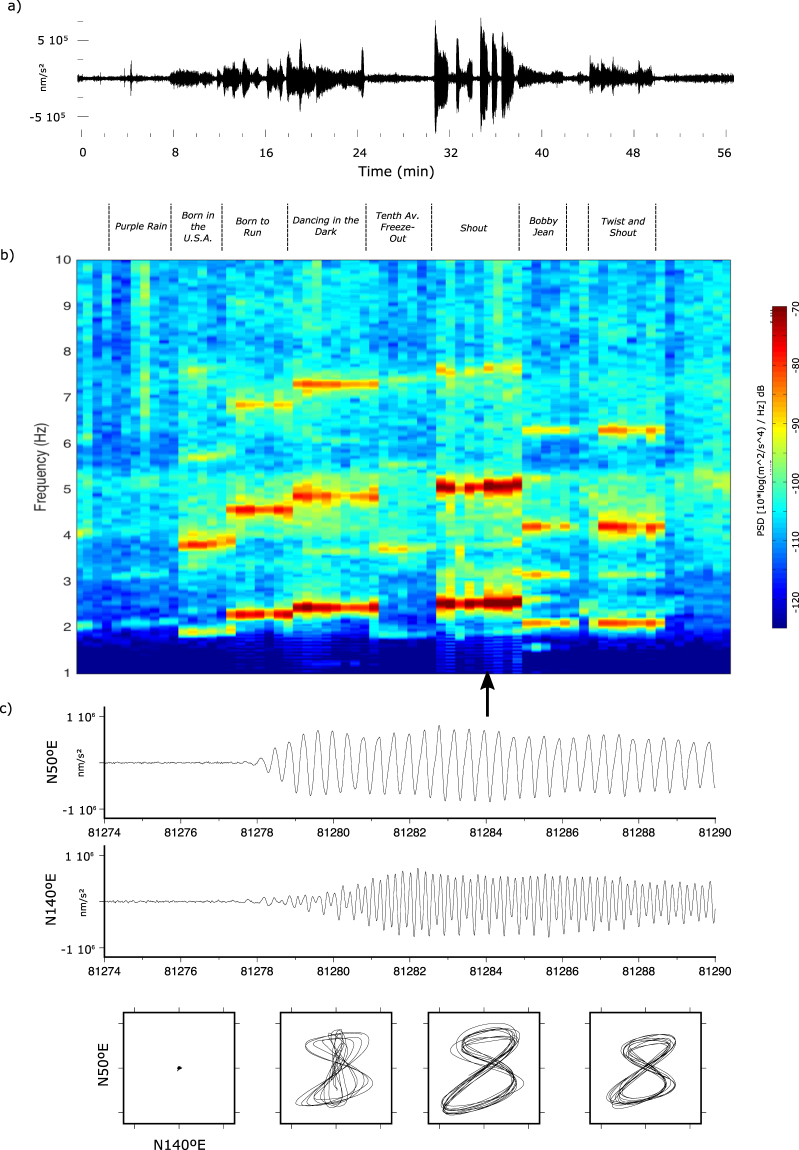
Seismic record during the “Encores” part of the Bruce Springsteen rock concert at FCB stadium (14/05/2016). (**a**) North-South component of the horizontal seismic acceleration Trace has been band-pass filtered between 1 and 10 Hz. (**b**) Corresponding spectrogram. Each song has a specific frequency content showing a fundamental frequency and harmonics. Titles of the songs are included for reference. (**c**) Particle motion of the horizontal components rotated to N50E/N140E during one of the Encores songs (marked by an arrow in the spectrogram). Each frame corresponds to 4 s. Some seconds after the beginning of the signal the phase shift between the fundamental and 1^st^ harmonic mode results in a PPM following a Lissajous curve.

We will refer here to the signals recorded during the Bruce Springsteen & E Street Band concert held in the FC Barcelona stadium 14^th^ May 2016. Up to 65000 persons fulfilled the stadium during the more than 3 hours of concert. The seismic signal generated during the concert is clearly visible in the three components, although the maximum amplitudes are observed in the horizontal components. The recorded seismic amplitudes show very large variations, often within the different parts of a single song (Fig. 5a). The spectrogram shows how each song results in a characteristic spectra composed by narrow and evenly spaced peaks, corresponding to a fundamental tone in the range of 1.8 to 2.5 Hz and two or three additional harmonics (Fig. 5b). Signals dominated by discrete vibration frequencies have been observed in seismic data from natural environments, including unrest volcanoes^34^, colliding tabular icebergs^35^ or hydrothermal sites^36^ and have been interpreted as resulting from regular repeating stick-slip earthquakes through the Dirac comb effect, stating that series of evenly spaced pulses in the time domain transforms to evenly spaced harmonics in the frequency domain^37^. This hypothesis can be explain our observations, as the synchronized jumps of the audience at a given rhythm can be assimilated to the occurrence of regular repeating earthquakes.

The fundamental tone frequency range is consistent with the previous results and with laboratory measurements of the movement of people dancing concluding that large groups tend move with frequencies in the 1.8–2.3 Hz frequency range^38^. The seismic amplitudes are strongly increased during the Encores, and, in particular, during the “Shout” song (Fig. 5), while the audio band of the concert do not show an increase of the sound volume. Table 1 presents the tempo of the songs played during the Encores, measured in beats per minute (BPM), and the fundamental frequency and harmonics of the corresponding seismic signal as identified in the spectrogram. The BPM values refer to recorded versions of the songs and not to the actual version played on this particular show and hence a formal correlation between BPM and fundamental frequencies can not be established properly. However, it seems clear from the data that fast songs (Born to Run, Dancing in the Dark) have higher fundamental frequencies, while slow songs (Tenth Av., Born in the USA) have lower values.

**Table 1:** Set list for the Encores part of the Barcelona 14/05/2016 Bruce Springsteen and the E-Street Band concert.

Song Title	Duration	Fundamental Frequency (Hz)	1^st^ harmonic (Hz)	2nd harmonic (Hz)	3th harmonic (Hz)	BPM
Purple Rain	6:01	2.1	—	—	—	113
Born in the U.S.A	4:43	1.9	**3.9**	5.7	7.6	122
Born to Run	6:35	**2.2**	4.5	6.8	—	146
Dancing in the Dark	7:00	**2.4**	4.9	7.4	—	149
Tenth Avenue Freeze-Out	5:21	**1.9**	3.7	5.5	7.4	117
Shout	6:46	**2.5**	5.1	7.6	—	139
Bobby Jean	6:12	2.1	**4.2**	6.4	—	133
Twist and Shout (tour debut)	6:56	2.1	**4.2**	6.4	—	131

Song duration, observed frequency peaks for the fundamental and harmonic modes and the beats per minute of recorded versions of the played songs are included. Bold numbers show the harmonic with maximum amplitude. BPM data recovered from http://songsbpm.com.

Hotovec *et al*.^37^ explained the smooth increase of the fundamental and the harmonics frequencies observed in the Redoubt volcano by an increase in the pace of earthquake occurrence. This hypothesis is consistent with the relationship between fast songs and high seismic frequencies observed in our data and provides additional support to the hypothesis that dancing crowd is on the origin of the observed seismic signal, which dominant frequency is directly controlled by the music rhythm.

Particle motion diagrams provide and additional tool to investigate this signal, showing that the signal during the songs has a mean polarization oriented N50°E, close to the transverse direction between ICTJA and the music stage. During the bursts of maximum seismic amplitude (“Shout” song, Fig. 5) the horizontal particle motion diagrams show a pattern very similar to a Lissajous curve with a ratio factor of 2 and a phase difference close to 90° (Fig. 5c). For intermediate amplitudes (e.g. during “Born in the USA” or “Dancing in the Dark” songs) the particle motion diagrams show patterns similar to Lissajous curves with frequency ratios of 2 and phase shifts of 20–30°, while for smaller amplitude events no particular features are observed. These particle motion diagram results from the fact that most of the seismic energy is concentrated near the fundamental and first harmonic modes (2.54 and 5.1 Hz in this case). Filtering the signal around those frequency peaks it can be observed that the fundamental mode is oriented N60°E and shows some ellipticity during the first seconds, while the first harmonic is linearly polarized and oriented N50°W. The characteristics of the different modes change for the different segments analyzed so far, but phase shifts remain close to 100°. Figure 5c allows also to note that the phase shift relationship is only settled after the first 2–3 s of shaking. This is consistent with^30^, who reported that it takes about 15–20 load cycles to build up a steady state response of the ground following the beginning of a synchronized movement (dancing) of the audience. Hereof, it seems that during interval of large shacking, and after a short initial time, the fundamental and first harmonic acquire a phase shift which remain constant during each bursts but change for each episode.

Beside propagating as seismic waves in the soil, the vibrations generated by dancing crowd can also excite some of the stadium structures. These structures are difficult to model accurately, but their excitation frequencies usually ranges between 1.0 to 3.5 Hz and 2.8 Hz^39^. Building codes define natural frequency limits for structures subjected to dynamic loading, which is fixed around 6–8 Hz (ref.^39^ and references hereby). It is worthy to note that the larger amplitudes during the concert have been observed during the song with the highest fundamental frequency value, with its first harmonic relatively close to the suggested limit for natural frequencies. Additional work, following a more engineering approach, is required to know if structure excitation has a significant contribution to the total shaking.

### Seismic record of fireworks

**Figure 6:**
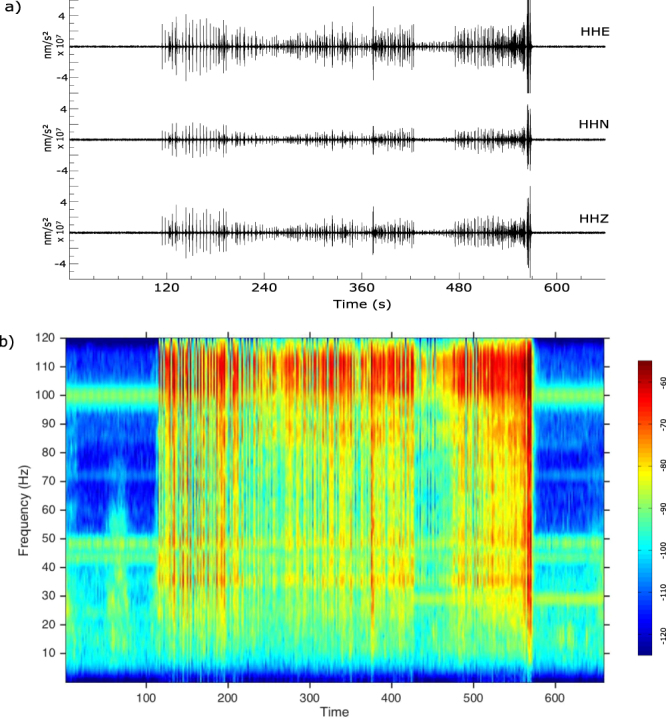
Seismic record of a fireworks show at the FC Barcelona stadium (7/6/2015). Firework exhibition carried on in the FC Barcelona stadium the 14^th^ June 2015 during the celebration of the football titles won in the 2014–15 season. Single explosions during the firework exhibition can be identified in the seismic record, with duration close to 1s. The amplitude of each shot is typically between 2.5 10^7^ and 4 10^7^ nm/s^2^, but peak values of 1.2 10^7^ nm/s^2^ are reached during the last part of the show. (**a**) Three components of the seismic acceleration during the 12 minutes interval including the fireworks. (**b**) Spectrogram of the vertical component of acceleration.

Firework exhibitions carried on in the FC Barcelona stadium during football title celebrations generate seismic signal clearly recorded at ICTJA station (Fig. 6). The signals have a very large spectral content, extending from frequencies below 0.01 Hz to the upper limit of our analysis capability (125 Hz), although most of the energy is comprised at frequencies exceeding 20 Hz. The stronger energy is identified in the final part of the records, correlating with the strong explosions that typically mark the end of firework shows. In the 20–80 Hz pass band the energy is distributed rather uniformly, although energy increases are detected around 35 Hz and 48 Hz. Between 80 and 100 Hz the energy start to increase to reach a maximum between 100 Hz and 125 Hz (Fig. 6b). The vertical and the North-South components have similar amplitudes, while the East-West has about 3 times higher amplitude. The horizontal polarization have a mean value close to N75E, although energy is detected in the N65E-N105E azimuth range.

Comparing the seismograms during firework with those recorded during the music concert, we can note that significant differences do appear. The energy of the waves recorded during firework extend over the whole spectrum and can be identified in the raw signal, while during the music event, the signal is less energetic, limited to the 2–6 Hz band and distributed in harmonics. During the concert the amplitude of the vertical component is lower than in the horizontal ones, while during fireworks the amplitudes of all the components are similar. Additionally, the signal generated by the firework explosions have linear particle motion diagrams, in contrast with the particle motion following a Lissajous curve recorded during the music concert. We interpret that, as the explosions during fireworks occurs on air, the most plausible origin for the detected signals is the acoustical-mechanical conversion of the sonic waves generated at each explosion. This distinct mechanism will explain the large differences between vibrations related to fireworks and crowd dancing, even if the source location is the same. It is well known that acoustic waves can couple to the ground and propagate as seismic waves and it has been documented how nuclear and chemical explosions in the atmosphere can generate seismic waves recorded at long distances [e.g., ref.^40^]. Although we have not found references on the seismic recording of fireworks, it seems plausible to record seismically that such kind of explosions at distances around 500 m. Edwards *et al*.^41^ analyzed acoustic-seismic coupling of meteor shock waves in a site located over a 60 m thick silt-clay layer and found that the coupling was efficient for frequencies above 10 Hz, consistently with our observation of seismic energy mostly above 10–20 Hz. A full understanding of such signals seems however difficult to model, as air-ground wave conversions can occur in any place around the area and the response of each building would need to be taken into consideration.

### Footquakes

Seismic recording of people moving to celebrate goals or relevant plays during sport events have been reported episodically, both in soccer and American football. A college American football game played in Louisiana on October 1988 (https://en.wikipedia.org/w/index.php?title=Earthquake_Game&oldid=747160712) was recorded seismically and is since then know as the “Earthquake Game”. More recently, a game between the Seahawks and the New Orleans Saints recorded in Seattle on January 2011 was named the “Beast Quake”^42^ and motivated the installation of a network of seismic instruments in the Seahawks stadium, managed by the Pacific Northwest Seismic Network (https://www.pnsn.org/seahawks). This approach seems to be extending, as shown by the FanQuakes project, carried on by the Ohio Geological Survey and the Miami University to measure the movement of fans during games at Ohio Stadium (https://sites.google.com/a/miamioh.edu/brudzinski/fanquakes).

In the case of soccer, the first reported example of seismic records of fans celebrations is the so-called “Gol del terremoto” (Earthquake’s Goal), recorded in 1992 by a seismometer of the Observatorio Astronómico La Plata (Argentina), located about 600 m away from the football stadium. (https://es.wikipedia.org/w/index.php?title=Gol_del_terremoto&oldid=95847086). During a temporary broad-band deployment in Cameroon in 2006, tremor like signals recorded simultaneously all around the country surprised a research team carrying on a seismic exploration project in the country and were finally attributed to the lively celebrations of people watching television broadcasts of the National Football Team in the 2006 African Cup of Nations^43^. Recent examples of seismic records of crowds celebrating soccer goals were presented last year by the University of Leicester and the British Geological Survey and had a significant impact in social networks, including a dedicated Twitter account (@Vardyquake).

**Figure 7:**
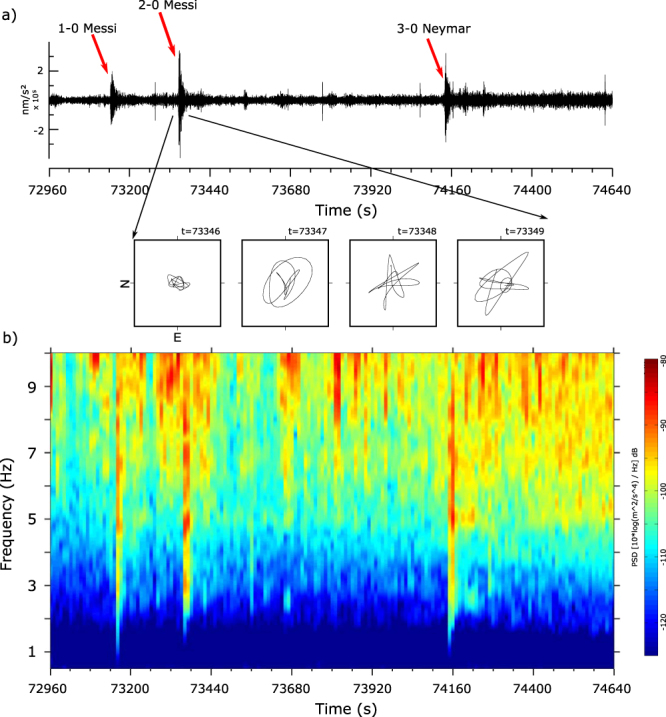
Seismic record during the last minutes of the FC Barcelona vs. FC Bayern Munich (Champions League semi-finals, 6/5/2015). (**a**) Vertical acceleration band-pass filtered between 2 and 6 Hz. Earth shaking following FCB goals is shown by red arrows. Lower panels show the particle motion diagram of the horizontal components during the onset of the second goal (1 s per panel) (**b**) Spectrogram of the same time interval. No harmonics modes are observed in this case.

The ICTJA station regularly record this kind of “footquakes” generated by people shaking to celebrate goals at the FC Barcelona stadium, hosting up to 90000 persons during first level games. We analyze here the records corresponding to the celebrations during the Champions League Semi-Finals game FC Barcelona – Bayern Munich played in May 2015, as they may provide some clues to the interpretation of sources of the different features described above. The three goals scored by the FC Barcelona team concentrate in the last 15′ of the game, thus providing a nice seismogram record (Fig. 7a). The recorded signals are observed clearly in the 1–6 Hz band, in a very similar range that those recorded during music events. The onset of the three events analyzed has an emergent character and their duration is very similar, 17–18 s. The amplitude of the peak accelerations differs in the three cases, the second one being that with largest amplitude, reaching values of +/−3.5 10^5^ nm/s^2^ for the East-West component. In all the cases the amplitude of the horizontal components is about 3 times larger than in the vertical one. The particle motion diagrams do not show a well-defined polarization but a detailed inspection of the onset of each event shows an elliptical motion in the horizontal plane, suggesting the interaction of multiple wavefronts (Fig. 7a). Most of the energy arrive with backazimuthal direction close to N50E, in the approximate transverse direction of propagation. The small amplitude of the vertical component and the dominant energy along the transverse direction of the horizontal components seems to discard a propagation as Rayleigh wave.

The major difference with the music concert case arises from the analysis of the frequency content of the signals. As shown at Fig. 7b, the seismic energy in this case is distributed quite uniformly along the 1–10 Hz band, without the presence of harmonics. This fact is interpreted as reflecting the characteristics of the crowd movement of during a goal celebration, when people jumps suddenly from their seats and remain moving on place for some seconds. This sudden jump of up to 90000 people (typical assistance range at FC Barcelona stadium) results in a sudden vibration, but it is not enough to build a steady vibration resulting in well-defined harmonics. This is consistent with previous reports pointing that several load cycles are required to build such a steady vibration state^30^.

## Conclusions

The seismic records provided by a broad-band seismometer in an urban environment provides a powerful outreach tool, as general public and mass media use to be interested in those kind of curious features. This interest can be used to disseminate some aspects of the seismological research to wide audiences. Besides this point, a detailed inspection of the recorded data reveals some intriguing aspects which may be of interest for a better understanding of unusual sources of seismic signals. A broad-band seismometer has revealed to be a useful instrument to monitor the traffic along an avenue located at about 150 m distance. Although mechanic devices or accelerometers are much less expensive instruments to monitor traffic, in some specific circumstances the use of high sensitivity broad-band instruments can be considered, as it does not need to be installed in the close vicinity of the monitored road, it is a robust instrumentation, can be installed in a secured location, can provide real time signal and allows to image graphically the traffic level.

This monitoring ability extends to the surveying of subway trains, which tunnel runs beneath the same main avenue at 150 m of our site. At frequencies around 30 Hz, the metro noise can be isolated from the traffic generated signal, hence allowing the identification of single train passages. Subway activity can also be clearly detected at very low frequencies (0.005–0.1 Hz) in a range not explored in engineering surveys. The origin of this low frequency signals has to be investigated in further detail, as it could be related to ground deformation during the train passages or to the effect of the magnetic field variations due to leakage currents. In this moment we privilege this last hypothesis, although more theoretical work is needed to understand how the magnetic field variations induced by those stray currents are recorded by broad-band seismometers. The seismic signals recorded during fireworks are very different from the rest of events here analyzed, supporting the idea that they correspond to a acoustic-mechanic wave coupling. Seismic signals recorded during music concerts and football events differs in their frequency content; during goal celebrations energy is uniformly distributed between 1 and 6 Hz, while during the music concert harmonic modes are clearly recognized and varies even within a single song. These differences are evidenced in the particle motion diagrams, showing a simple elliptical motion for goal celebrations and Lissajous curves during music concert. We think that both for music concerts and football goal celebrations the origin of the seismic signal is the movement of the people jumping or dancing altogether, hence generating vibrations on the ground and the structures of the stadium that then propagate as seismic waves. The coordinated movement of people dancing following the music tempo results in the building of a steady response of the ground resulting in harmonic seismic signals similar to those observed in some volcano environments. The particle motion diagrams show that 2–3 seconds are needed to build this response following the beginning of the crowd movement. On contrary, the celebrations after football (soccer) goals, not exceeding 20 s, generate a significant amount of shaking but do not result in harmonics. In this sense, different studies have show that a coordinate movement of a crowd is more easy when people is prompted my music stimuli, a point that seem to be confirmed by seismic data. We can conclude that urban seismology can be used not only to discriminate among the different sources of vibrations in urban environments, but also to better understand the multiple mechanisms involving the seismic signal generation, including features as diverse as magnetic field variations, acoustic-mechanic coupling or resonances in buildings.

## Methods

The instrument response is removed from the raw seismic data using the standard procedures and the signal is converted to acceleration.

Data is analyzed using the PQLX software package^44^ to estimate systematically the background noise Power Spectral Density (PSD) and to compute Probability Density Functions (PDFs). PDFs provide a useful tool to analyze the energy distribution across the seismic spectrum and to monitor its temporal variations. The model typically used as a reference to assess the quality of new seismic sites is that of Peterson^45^, defining the standard New Low and High Noise level Models (NLNM, NHNM). Background noise in new seismic sites areas is expected to lay below NHNM across the spectra.

Spectrograms are obtained by dividing the signal in overlapping intervals of a given length and calculating the Power Spectral density in each of them. In this case we have used an own MatLab routine based on the Pwelch algorithm. A color palette, expressed in dB and relative to a reference value of 1 (m^2^/s^4^)/Hz, is used to show the energy distribution.
